# Unveiling Cs-adsorption mechanism of Prussian blue analogs: Cs^+^-percolation *via* vacancies to complete dehydrated state†

**DOI:** 10.1039/c8ra06377j

**Published:** 2018-10-10

**Authors:** Akira Takahashi, Hisashi Tanaka, Kimitaka Minami, Keiko Noda, Manabu Ishizaki, Masato Kurihara, Hiroshi Ogawa, Tohru Kawamoto

**Affiliations:** Nanomaterials Research Institute, AIST 1-1-1 Higashi Tsukuba 305-8565 Japan tohru.kawamoto@aist.go.jp; Department of Material and Biological Chemistry, Faculty of Science, Yamagata University 1-4-12 Kojirakawa-machi Yamagata 990-8560 Japan; Research Center for Computational Design of Advanced Functional Materials, AIST 1-1-1 Umezono Tsukuba 305-8568 Japan

## Abstract

Metal hexacyanoferrates (MHCF) or Prussian blue analogs are excellent Cs^+^-adsorbents used for radioactive Cs-decontamination. However, the adsorption mechanism is controversial. To clarify the issue, we quantitatively investigated the Cs-adsorption behaviors of potassium copper hexacyanoferrate (KCuHCF) and A_*y*_Cu[Fe(CN)_6_]_1−*x*_·*z*H_2_O. To obtain samples having homogeneous chemical composition and particle size, flow systems were used for both synthesis and purification. After sufficient rinsing with water, the range of *x* stable in aqueous solution in time appropriate for Cs-adsorption was 0.25 < *x* < 0.50. The relations *y* = 4 − 2*x* and *z* = 10*x* were also found independent of *x*, indicating complete dehydration of K^+^ in the crystal. We concluded that the excellent Cs-selectivity of MHCF was not due to difference in free energy of the adsorbed state between K^+^ and Cs^+^ but because of the hydrated state in aqueous solution. We also found that the guiding principle for determining the maximum capacity depended on the chemical composition. In particular, for the range 0.25 < *x* < 0.35, we propose a new model to understand the suppression of the maximum capacity. In our model, we hypothesize that Cs^+^ could migrate in the crystal only through [Fe(CN)_6_]^4−^ vacancies. The model reproduced the observed maximum capacity without fitting parameters. The model would also be applicable to other MHCFs, *e.g.* a little adsorption by soluble Prussian blue. The ion exchange between Cs^+^ and H^+^ occurred only when the implemented K^+^ was small.

## Abstract 2

Cs^+^ adsorption selectivity caused by hydration energy and percolation theory to evaluate the ion-exchangeable site ratio.

## Graphical abstract

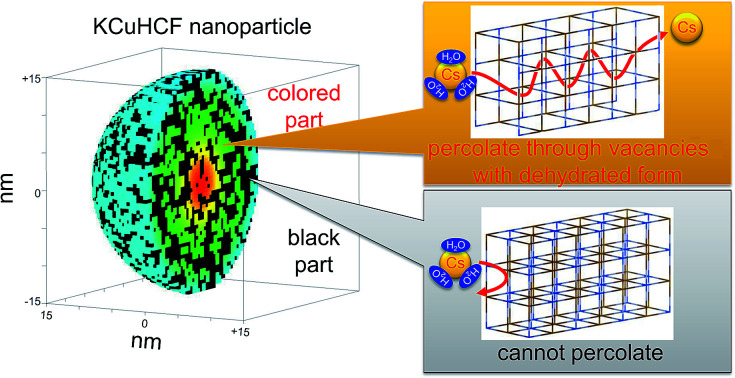

## 1. Introduction

When accidents happen due to radioisotope leakage from atomic power plants, contamination of the environment by radioactive cesium is often serious.^1,2^ Cs-137 particularly shows a moderate half-life time of 30.2 years, resulting in large radioactivity and long influence. During waste liquid treatment at nuclear facilities, Cs-137 creates large problems because its high solubility in water causes the spread of contamination. Cs-137 also contributes to the heat load. The volume of high-level radioactive waste can be reduced by separating Cs-137.

For the separation and decontamination of radioactive caesium, the use of adsorbent is efficient and cost effective. Among various adsorbents, metal hexacyanoferrate (MHCF),^3–12^ zeolite,^13–15^ calix arenes,^16–18^ ion exchange resins,^19^ metal organic frameworks,^20^ ionic crystal,^21^ and silicotitanate^22,23^ have been surveyed and used as Cs-adsorbents. Metal hexacyanoferrates are known for high selectivity, capacity, and applicability for trace concentrations of cesium.

MHCF is a porous coordination polymer, consisting of metal cations M^α+^ and hexacyanoferrate anions, [Fe(CN)_6_]^β−^. A historical pigment, Prussian blue (PB), A_*y*_Fe[Fe(CN)_6_]_1−*x*_·*z*H_2_O, is one of the MHCFs. MHCF has a porous network in its crystal, resulting in potential application for the adsorbent or storage of small molecules^24–29^ and ions.^30–34^

Although MHCFs have already been put to practical use, their adsorption mechanism remains unclear. The main adsorption mechanism is ion-exchange between Cs^+^ and other cations such as K^+^ and Na^+^ implemented in synthesis. However, there are some controversial properties. For example, it was reported that soluble Prussian blue, (NH_4_)_0.70_Fe_1.10_[Fe(CN)_6_]·1.7H_2_O, showed less equilibrium adsorption capacity than insoluble Prussian blue, Fe_4_[Fe(CN)_6_]_3_ in spite of increased incorporated cations.^8^ The adsorption by insoluble Prussian blue can be explained by ion-exchange between Cs^+^ and H^+^.^8,35^ However, why soluble Prussian blue adsorbs less Cs^+^ has not been revealed.

A couple of other adsorption mechanisms have been suggested concerning potassium copper hexacyanoferrate, KCuHCF, and related compounds. For example, Cu[Fe(CN)_6_]_0.5_ showed Cu^+^ elution after long adsorption, implying exchange between Cs^+^ and Cu^2+^.^36^ On the other hand, K_2_CuFe(CN)_6_ adsorbed Cs^+^ with degradation of the crystal structure.^37^ Thus, unclear issues for the adsorption mechanism of MHCF remain. The main purpose of this paper is to unveil the adsorption mechanism. For the purpose, we focus on KCuHCF because it can be synthesized with variation in its composition ratio, which would help us understand the composition dependence on Cs^+^ adsorption performance. In contrast, it is difficult to prepare PB in a variety of compositions.

KCuHCF is preferred for environmental decontamination because of its high performance at low cost.^5,38–43^ The Cs-capacity of KCuHCF is as high as 2.5 mmol g^−1^.^10^ Its selectivity is also sufficient, *e.g.*, it is used for radioactive caesium recovery from seawater for trace monitoring.^44^ The elution of CN^−^ at neutral condition was suppressed relative to PB.^5^

**Fig. 1:**
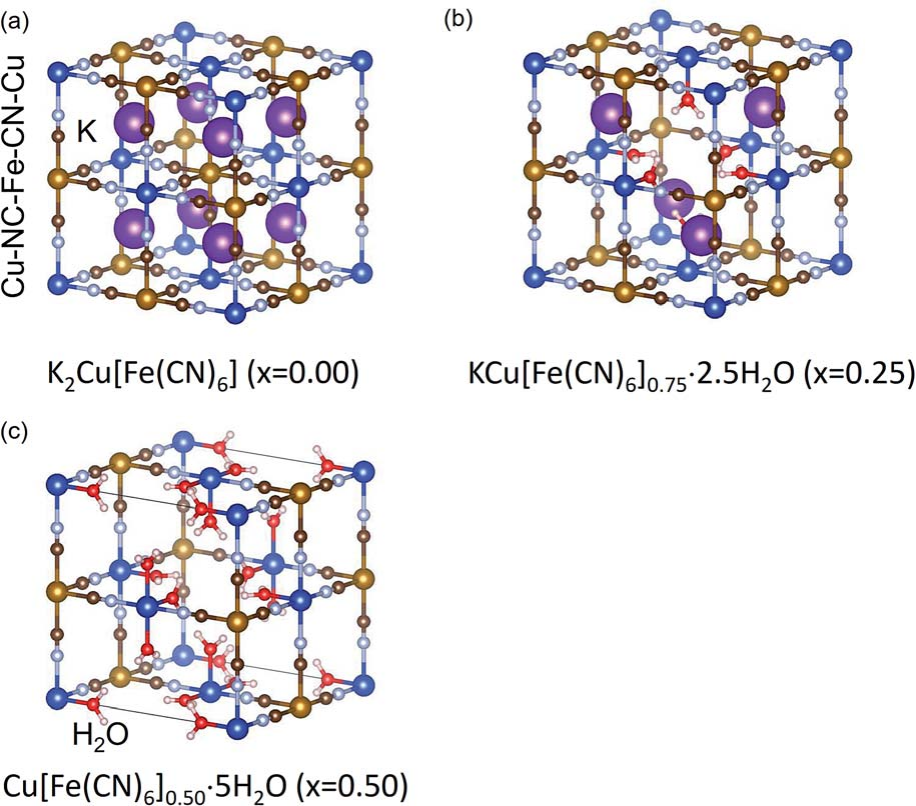
Schematic view of expected crystal structures of potassium copper hexacyanoferrate, KCuHCF, at *x* = 0.00, 0.25, and 0.50. H_2_O molecules at interstitial sites are omitted for simplicity.

Schematic views of the crystal structure of KCuHCF are shown in Fig. 1. In MHCF, there are two kinds of pores in the crystal. One is the interstitial site surrounded by a cubic cage consisting of eight metals bridged by twelve cyano-groups. The other includes vacancy sites, implemented by the introduction of [Fe(CN)_6_] vacancies. By controlling the ratio of [Fe(CN)_6_] vacancies, the chemical composition of KCuHCF is described as K_*y*_Cu[Fe(CN)_6_]_1−*x*_·*z*H_2_O, where *x* represents the [Fe(CN)_6_] vacancy ratio. Examples of the crystal structures of KCuHCF at *x* = 0.00, 0.25, and 0.50 are shown in Fig. 1. At a [Fe(CN)_6_] vacancy, six H_2_O molecules are coordinated with surrounding Cu^2+^ cations. The [Fe(CN)_6_] vacancies are randomly distributed in the crystal.

At first, we evaluated the range of chemical composition stability in aqueous solution. Using stable materials in our Cs^+^-adsorption test, we excluded the elution of ions from KCuHCF unrelated to ion-exchange with Cs^+^. Although the stability of KCuHCF was quite high due to extremely low solubility product, p*K* = 37.8 for K_0.66_Cu[Fe(CN)_6_]_0.66_,^45^ the elution of consisting ions should be avoided. For example in Japan, the standards of Cu^2+^ and total CN in effluent are 3 mg L^−1^ and 1 mg L^−1^, respectively.^46^

In this paper, we used flow techniques to reduce the fluctuation of sample characteristics such as chemical composition and size of nanoparticles. In addition, in this paper, a cross-flow filtering system was also used for constant and quantitative purification. It was only possible to discuss the adsorption mechanism with sufficiently quantitative preparation of samples.

One of the main aims of this paper is to clarify the origin of the excellent selectivity of Cs^+^ for metal hexacyanoferrate. Hence, we precisely evaluated the chemical composition with various [Fe(CN)_6_] vacancy ratios. From the result, we concluded that the alkali cations stayed in interstitial sites in a completely dehydrated form and that complete dehydration was the main reason for selectivity.

The other aim was quantitative understanding of the relationship between Cs^+^-maximum capacity and chemical composition. The obtained adsorption capacity cannot be explained only with a simple ion-exchange mechanism between K^+^ and Cs^+^. To explain the same, we propose a new model based on percolation theory in which only the adsorption site having connectivity from the surface of the KCuHCF particle through [Fe(CN)_6_] vacancy sites can adsorb caesium.

## 2. Experimental method

### 2.1. Synthesis and stability test

**Fig. 2:**
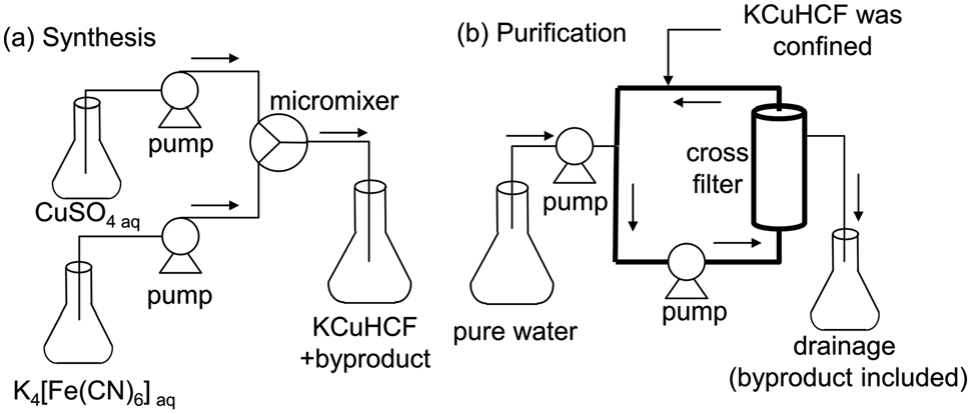
Schematic views of the flow systems for the preparation of KCuHCF-NPs. (a) Micromixer system for the synthesis of KCuHCF-NPs, and (b) cross-flow filter system for the purification of KCuHCF-NPs slurry. The thick line represents the circulation path where KCuHCF-NPs were confined.

To synthesize KCuHCF nanoparticles(NPs), the flow mixing using micromixer shown in Fig. 2(a) was used.^10^ KCuHCF-NPs was synthesized by mixing an aqueous solution of CuSO_4_ with that of K_4_[Fe(CN)_6_] using a micromixer with 150 μm hole diameter. The flow rate was set to 50 mL min^−1^ for each solution. The concentration of K_4_[Fe(CN)_6_] was fixed at 100 mmol L^−1^, whereas that of CuSO_4_ was varied in the range 100–500 mmol L^−1^ to control the chemical composition of KCuHCF-NP. In Table 1, the variation of mixing ratio, *R*_mix_ = Cu/[Fe(CN)_6_], is described. *R*_mix_ was varied from 1.00 to 0.20. The [Fe(CN)_6_] vacancy ratios expected from *R*_mix_, defined as *x*_exp_, are also shown in Table 1, where *x*_exp_ is calculated with the assumption that all raw materials in the solution were completely used in the synthesis.

**Table 1:** Synthesis conditions and structures of obtained KCuHCF-NPs. *R*_mix_, *x*_exp,_ and *x*_obs_ represent the molar ratios of K_4_[Fe(CN)_6_] to CuSO_4_ when mixing using the micromixer, [Fe(CN)_6_] vacancy ratio *x* expected from *R*_mix_, and that observed by chemical composition analysis, respectively

	Synthesis condition		Observed properties		
	*R*_mix_	*x*_exp_	*x*_obs_	Chemical composition	a (Å)
KCF0.00	1.00	0.00	0.25	K_1.00_Cu[Fe(CN)_6_]_0.75_·2.3H_2_O	10.05
KCF0.17	0.83	0.17	0.25	K_1.00_Cu[Fe(CN)_6_]_0.75_·2.4H_2_O	10.04
KCF0.29	0.71	0.29	0.30	K_0.77_Cu[Fe(CN)_6_]_0.70_·2.8H_2_O	10.03
KCF0.33	0.67	0.33	0.33	K_0.70_Cu[Fe(CN)_6_]_0.67_·3.3H_2_O	10.03
KCF0.38	0.62	0.38	0.38	K_0.47_Cu[Fe(CN)_6_]_0.62_·3.9H_2_O	10.02
KCF0.41	0.59	0.41	0.41	K_0.33_Cu[Fe(CN)_6_]_0.59_·4.1H_2_O	10.00
KCF0.44	0.56	0.44	0.43	K_0.19_Cu[Fe(CN)_6_]_0.57_·4.4H_2_O	9.99
KCF0.50	0.50	0.50	0.48	K_0.05_Cu[Fe(CN)_6_]_0.52_·4.6H_2_O	9.96
KCF0.67	0.33	0.67	0.50	K_0.00_Cu[Fe(CN)_6_]_0.50_·4.8H_2_O	9.97
KCF0.80	0.20	0.80	0.50	K_0.00_Cu[Fe(CN)_6_]_0.50_·4.7H_2_O	9.99

For purification, we made a rinse of KCuHCF-NP slurry in Milli-Q water with the system shown in Fig. 2(b), where the KCuHCF slurry was confined in the circulation path. Milli-Q water was supplied at the same rate as the filtration flow rate. After sufficient introduction of pure water, the rinsed KCuHCF was recovered from the circulation path. The system was based on the Cs-concentration system DBW-24 (Oct Science corp.) with UF-type hollow fibre filter (Microza AHP-1010, Asahi-Kasei Chem. Corp.). The filtration rate was set at 50–100 mL min^−1^. To achieve sufficient rinsing, the rinse was continued until the conductivity of the filtrate was below 50 μS cm^−1^. Subsequently, KCuHCF-NP powder was obtained by evaporation at 60 °C.

For a more severe test of stability in aqueous water, we used the same system as used for the desalination of the KCuHCF-NP slurry, as shown in Fig. 2(b). With *R*_mix_ = 0.65 (*x*_exp_ = 0.35) as the most stable condition, KCuHCF was prepared by mixing 155 mmol L^−1^ K_4_[Fe(CN)_6_] solution with 100 mmol L^−1^ CuSO_4_ solution to obtain Cu/Fe = 0.65. For the test, the prepared KCuHCF was estimated to be 6.7 g, and 38 L of Milli-Q water was used for rinsing. The liquid to solid ratio was 5670 mL g^−1^, 5.8 times larger than in the synthesis of KCF0.33 described above. The variance in chemical composition during rinsing by the cross-flow filter was investigated. The chemical composition was obtained both for the KCuHCF slurry and the dried powder. The concentrations of Cu, Fe, K, and SO_4_ in filtrate water were also evaluated. For Cu, Fe, and K, MP-AES was used. The valence number of Fe was checked by UV-Vis spectrometry (USB-4000, Ocean optics). SO_4_ concentration was evaluated with a water quality analyser (HS-2300, T&C Technical).

### 2.2. Characterization

The chemical compositions of KCuHCF-NPs were evaluated as follows: the composition ratio of K, Cu, and Fe was evaluated using decomposition of samples by microwave digestion (multiwave 3000, Perkin Elmer Inc.), followed by investigation of the concentration by inductively coupled plasma mass spectroscopy (ICP-MS, NexIon 300D, Perkin Elmer, Inc.). Water content was obtained by thermogravimetry (Thermo Plus EVO, Rigaku Corp.). The concentration of OH^−^ was estimated from charge balance in the chemical composition because part of H_2_O was sometimes converted to OH^−^ to satisfy charge balance.^47^

The crystal structure was identified by X-ray diffraction analysis (D2 Phaser, Bruker AXS Inc). The lattice constant was calculated by the Pawley method. Partly, we used the Rietveld method to determine the positions of alkali cations in the crystal. For angle calibration, the silicon powder NIST 640e was mixed in the samples as internal standard. The valence numbers of copper and iron were identified from the positions of the CN-vibration mode in FT-IR spectra measured by the diamond ATR method on iS5 (Thermofisher Scientific, Inc.). The images of KCuHCF-NP samples were obtained using a field-emission scanning electron microscope (FE-SEM, S-4800; Hitachi Hitec Corp.).

### 2.3. Cs-adsorption properties

The amounts of Cs adsorbed by KCuHCF samples and desorbed ions were evaluated by an adsorption test, *e.g.*, 10 mg of KCuHCF sample was added to CsNO_3_ aqueous solution with Cs-concentration of 300 mg L^−1^ and shaken at 25 °C using a shaker (SI-300C, AZ-ONE).

After shaking, the residue was separated from the supernatant. The concentration of Cs in the supernatant was evaluated by ICP-MS, and those of K and Cu by microwave plasma-atomic emission spectrometry (MP-AES, Agilent 4100). The amount of Cs adsorbed by the sample, *q*, was evaluated with the equation:

1 *q* = ([Cs]_ini_ − [Cs]_ad_) × (*V*/*m*),

where [Cs]_ini_ and [Cs]_ad_, represent the Cs-concentration in CsNO_3_ solution before and after adsorption, respectively. *V* and *m* respectively represent the solution volume and mass of KCuHCF sample.

The maximum capacity of KCuHCF, *q*_max_, was evaluated with a Langmuir plot fitted with the following equation,2
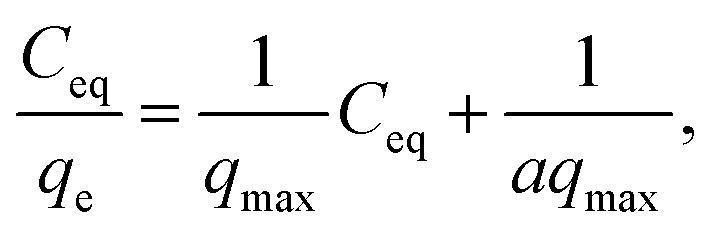
where *C*_eq_, *q*_e_, and *a* represent the Cs-concentration in an equilibrium state after mixing the solution in the solution, that in the adsorbent, and the equilibrium constant, respectively.

The shaking time was set to 100 min except for the case with *x*_obs_ = 0.50, which was determined by long time test up to 72 h for the evaluation of the time required to reach an equilibrium state. The shaking time dependence of the Cs adsorption amount is shown in Fig. SI1 (ESI†). When *x*_obs_ = 0.50, because the equilibrium state was not achieved even after 72 h of shaking, detailed analysis of the shaking time dependence of Cs adsorption amount and the variance in the chemical composition of KCuHCF were evaluated up to 72 h.

For the evaluation for the effect of the co-existent multiple ions, we also investigated the adsorption property with a solution including both Cs^+^ and Rb^+^ by adding CsCl and RbCl. The concentration of Cs^+^ and Rb^+^ were set to 2.26 mmol L^−1^ for both.

## 3. Results and discussion

### 3.1. Characterization of obtained KCuHCF-NPs

**Fig. 3:**
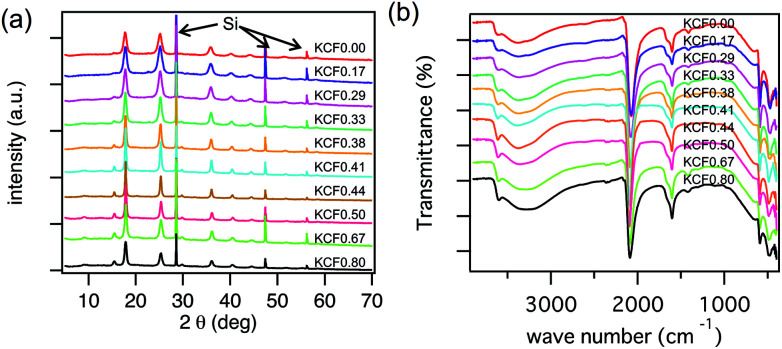
(a) XRD patterns of KCuHCF with different *R*_mix_, where *R*_mix_ represents the molar mixing ratio of CuSO_4_ to K_4_[Fe(CN)_6_]. The notation ‘Si’ indicates the diffraction peak corresponding to silicon powder mixed with the samples for angle calibration. (b) FTIR spectra of CuHCF with the variation of *R*_mix_.

**Fig. 4:**
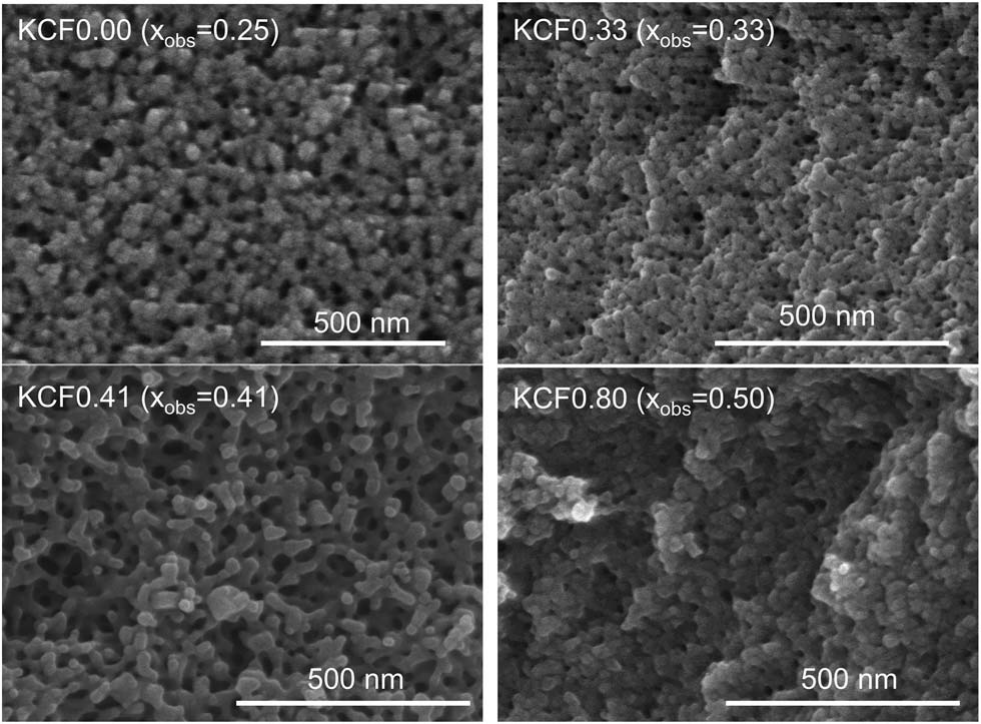
FE-SEM images of KCuHCF-NP samples with different *x*_obs_, the observed ratio of [Fe(CN)_6_] vacancies.

We evaluated the crystal structure using XRD. KCuHCFs synthesized with various *R*_mix_ showed the same crystal structure. Fig. 3(a) shows the XRD patterns, indicating that all samples had single phase with space group *Fm*3̄*m*. The lattice constant was almost same, in the range of 9.99–10.05 Å, but it became slightly longer as *R*_mix_ increased. This was caused by expansion of the lattice by K^+^ implementation into interstitial sites. SEM images of KCuHCF-NPs are shown in Fig. 4. The particle size seemed to be around 20–40 nm, independent of *R*_mix_.

FT-IR spectra indicated the valence numbers of Cu^2+^ and Fe^2+^ were retained in all samples, as shown in Fig. 3(b). The absorption peak corresponding to the CN-vibration mode was found at 2090 cm^−1^, almost the same as that in a previous report, 2095 cm^−1^.^48^

**Fig. 5:**
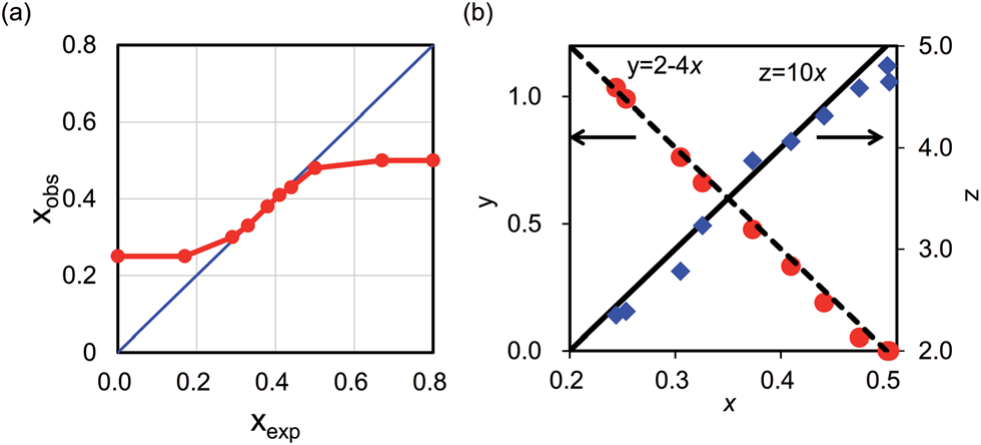
(a) Relationship between *x*_exp_ and *x*_obs_. (b) Relationships among the factors *x*, *y*, and *z* in chemical compositions of KCuHCF.

In contrast, the chemical composition varied depending on *R*_mix_, as shown in Table 1. The obtained composition almost satisfied charge balance, indicating that OH^−^ was not a main component unlike in potassium iron hexacyanoferrate.^47^ Slight difference from neutral was derived from the change in valence numbers of metals or from experimental errors. In some cases, the observed [Fe(CN)_6_] vacancy ratio, *x*_obs_, was different from the expected value, *x*_exp_. Table 1 and Fig. 5(a) show the difference between *x*_exp_, and *x*_obs_. When 0.30 ≤ *x*_exp_ ≤ 0.50, *x*_obs_ was almost the same as *x*_exp_. On the other hand, at *x*_exp_ ≤ 0.17 or *x*_exp_ ≥ 0.67, *x*_obs_ converged to *x*_obs_ = 0.25 and *x*_obs_ = 0.50, respectively. The results showed that the range of chemical composition that was stable in aqueous condition was 0.25 < *x* < 0.50. With this range of *x*, we also confirmed that the chemical composition was unchanged after the Cs-adsorption test described later.

**Fig. 6:**
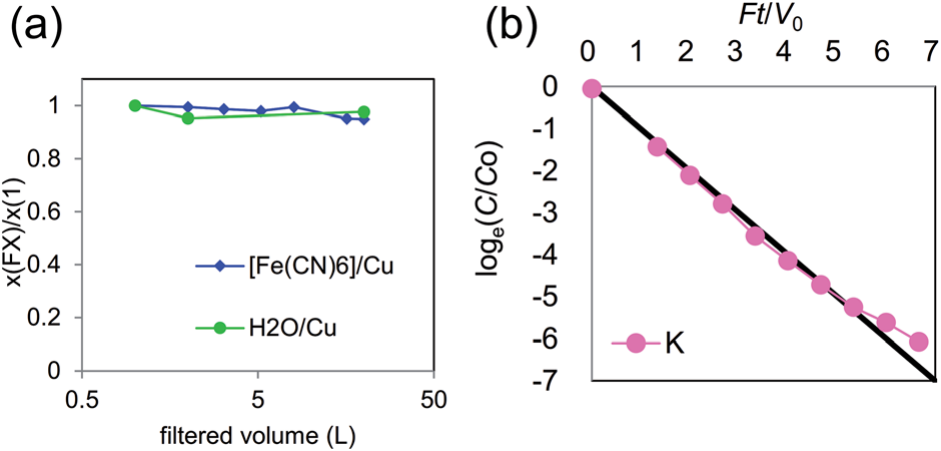
(a) Variance of chemical composition of KCuHCF-NP synthesized with *R*_mix_ = 0.65. (b) Ionic concentration in rinse solution obtained at the drainage of cross-flow filtering system.

To confirm stability, we investigated the change in chemical composition during further severe rinsing. In the case of *R*_mix_ = 0.65, the variances of the composition are shown in Fig. 6(a), where change in chemical composition was small. As shown in Fig. 6(b), the K^+^ concentration in rinse water decreased exponentially. This behavior can be explained by dilution due to the flow of rinse water, indicating that additional elution from KCuHCF during rinsing was limited. From these results, KCuHCF with *x* = 0.35 had sufficient stability in aqueous solution.

In the case of *x*_exp_ = 0.25, the chemical composition gradually changed to 0.65 during sufficiently long rinse, although the composition was constant both during synthesis and the Cs-adsorption test. This result indicated that the composition of *x*_exp_ = 0.25 was a metastable state. Concerning the case of *x*_exp_ = 0.50, a gradual change in the composition with Cu^2+^ elution was reported,^49^ but we observed no significant elution of Cu^2+^ in the Cs-adsorption test. Consequently, we concluded that the range 0.25 < *x* < 0.50 was stable for Cs-adsorption, although the composition may change because of long-time contact with water.

### 3.2. Complete dehydration of K^+^ in the lattice: the origin of excellent selectivity of Cs^+^

We evaluated the relationships in composition ratio among *x*, *y*, and *z*, as shown in Fig. 5(b). There were clear relationships, *y* = 2− 4*x* and *z* = 10*x*. These relationships could be understood as follows: *y*, the number of K^+^ in the unit-cell, could be calculated from the number to satisfy charge balance to cancel the effect of introducing [Fe(CN)_6_]^4−^ vacancies. The value *z*, the number of H_2_O, is calculated by the sum of six coordination water molecules around the vacancy site, 6*x*, and the number of interstitial sites unoccupied with K^+^, 2 − (2 − 4*x*) = 4*x*. This result indicated that each interstitial site could be occupied by only one K^+^ or H_2_O. The conclusion was also supported by Rietveld analysis with XRD patterns. With assumption of only one K^+^ or H_2_O occupation, the XRD patterns were well explained as shown in Fig. SI2 and Table SI1 in the ESI.†

Information on the occupancy of K^+^ and H_2_O was essential for Cs^+^ selectivity during adsorption by MHCFs because the relationship among *x*, *y*, and *z* indicated complete dehydration of alkali cations at the interstitial site in the KCuHCF crystal. High Cs-selectivity was caused by the complete dehydration of alkali cations at the interstitial sites. In the case of clay minerals, it is known that the removal of water from the interlayer enhances the selectivity of Cs-adsorption.^50^ This behaviour could originated from a change in the hydration number of K^+^ in MCHF. In MHCF, because K^+^ cations were located in a pseudo-dry environment separated from water molecules after adsorption, the Cs-selectivity of KCuHCF was strongly enhanced.

The mechanism was supported quantitatively by previous works. The alkali cations were found to be dehydrated completely. In other words, the hydration numbers of alkali cations were fixed at 0 after adsorption; hence, a quantitative discussion of adsorption energy is possible. The gain in free energy by ion-exchange between K^+^ and Cs^+^, Δ*G*, was calculated as:

3 Δ*G* = {*G*(K_hyd_) + *G*(Cs_ad_)} − {*G*(K_ad_) + *G*(Cs_hyd_)},

where *G*(A_hyd_) and *G*(A_ad_) (A = K or Cs), represent the free energy of A^+^ cation in the hydrated state and that of the state adsorbed into KCuHCF, respectively. Eqn (3) is decomposed into two components,

4 Δ*G* = {*G*(K_hyd_) − *G*(Cs_hyd_)} − {*G*(Cs_ad_) − *G*(K_ad_)}

5 Δ*G* = Δ*G*_hyd_ + Δ*G*_ad_,

where Δ*G*_hyd_ and Δ*G*_ad_ represent the difference in free energy of the hydrated state between K^+^ and Cs^+^, and that of the adsorbed state between K^+^ and Cs^+^, respectively. In the literature, *G*(K_hyd_) and *G*(Cs_hyd_) were reported to be −351.8 and −306.4 kJ mol^−1^, respectively,^51^ On the other hand, the adsorption energy Δ*G* was estimated at 69.087 kJ mol^−1^.^52^ From these values, Δ*G*_hyd_ and Δ*G*_ad_ are calculated at −45.4 kcal mol^−1^ and −23.7 kcal mol^−1^, respectively, indicating that the energy gain by Cs^+^-adsorption mainly came from the difference in the hydrated states between K^+^ and Cs^+^, and not from that in the adsorbed state.

For the confirmation of our consideration, we also evaluate the adsorption property of KCF0.33 with an aqueous solution including 2.26 mmol L^−1^ of both Cs^+^ and Rb^+^. To evaluate the selectivity between Cs^+^ and Rb^+^, such high-concentration was chosen, because both ions would be adsorbed from a solution with low concentration. The adsorption ratios are 66.4% for Cs^+^ and 36.2% for Rb^+^, respectively. The distribution coefficients are 1.8 × 10^3^ and 5.6 × 10^2^ mL g^−1^ for Cs^+^ and Rb^+^, respectively. The result indicates that KCuHCF can also adsorb Rb^+^, and that Cs^+^ was selectively adsorbed even from a solution having co-existing Rb^+^. It is consistent with the hydration energy or Rb^+^, −329.3 kJ mol^−1^,^51^ higher than that of Cs^+^ and lower than that of K^+^.

### 3.3. Cs-adsorption property

**Fig. 7:**
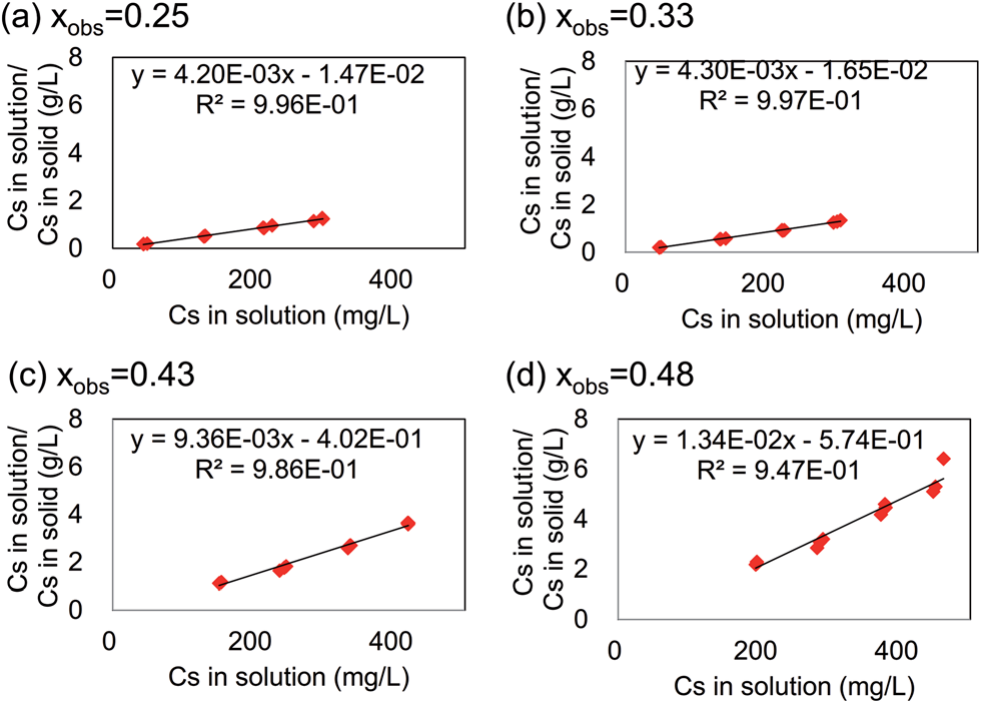
Langmuir plot for Cs adsorption by KCuHCF with varying *x*. The black line represents the fit curve by the Langmuir equation.

To clarify the relationship between the maximum capacity and the chemical composition, we did Cs^+^-adsorption tests for stable KCuHCF-NPs at 0.25 < *x* < 0.50. Fig. 7 shows examples of Langmuir plots for Cs adsorption by KCuHCF with various *x*. The plots for other *x* are shown in Fig. SI3 (ESI†). It was found that the Cs-adsorption behaviour by KCuHCF agreed with the Langmuir equation independent of *x*. However, the slope of the plot varied with changing *x*.

**Fig. 8:**
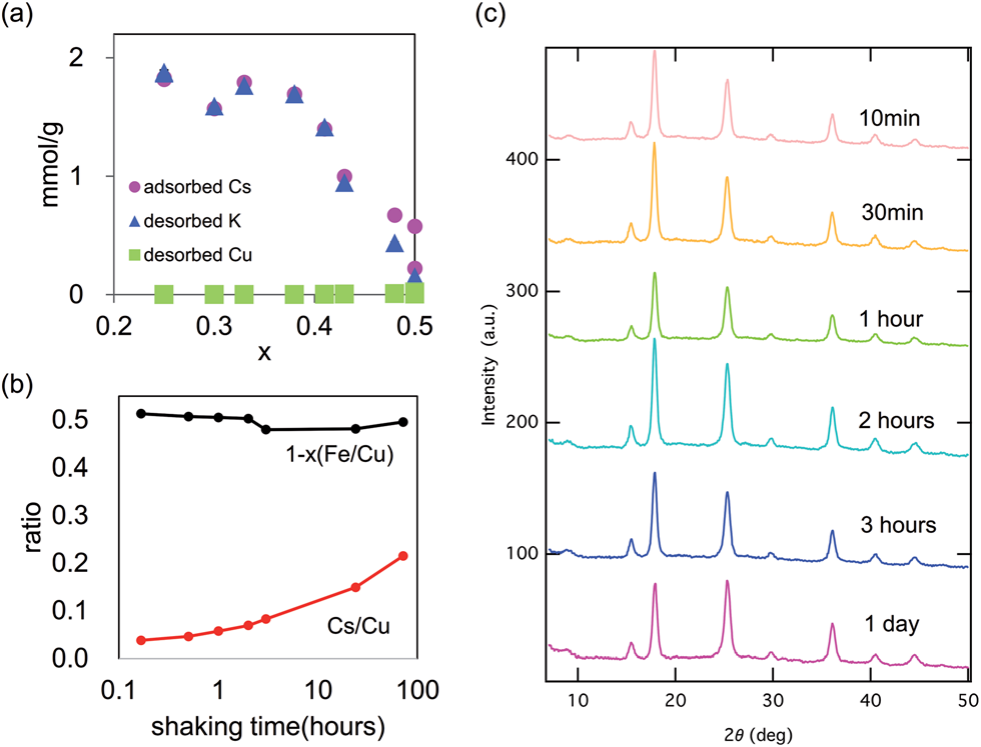
Cs-adsorption property of KCuHCF with various chemical compositions. (a) The amounts of adsorbed-Cs, desorbed-K, and desorbed-Cu, and (b) the variance in chemical composition of Cu[Fe(CN)_6_]_0.5_ during Cs-adsorption test. (c) The variance in the XRD patterns during shaking time in the Cs-adsorption test for KCF0.20 (*x*_obs_ = 0.50).

Fig. 8(a) shows the maximum capacity of KCuHCF evaluated by Langmuir plot analysis. The maximum value of saturated capacity *q*_max_ was 1.82 mmol g^−1^ (=242 mg g^−1^) at *x* = 0.25. However, in the range 0.25 < *x* < 0.38, *q*_max_ showed little dependence on *x*. At 0.41 < *x* < 0.50, *q*_max_ decreased as *x* increased. However, even at *x* = 0.50, a definite value of *q*_max_ was found. Fig. 8(a) also shows the desorbed amounts of K^+^ and Cu^2+^. In the range *x* < 0.4, the amount of desorbed K^+^ was almost equal to that of adsorbed Cs^+^. From the result, it was found that the main mechanism of Cs-adsorption in this range was ion-exchange between K^+^ and Cs^+^ in the range *x* < 0.4.

In contrast, at *x* > 0.4, the amounts of adsorbed Cs^+^ and desorbed K^+^ were different and the difference became larger as *x* increased. Even at *x* = 0.50, where no K^+^ was incorporated in KCuHCF (K_0.00_Cu[Fe(CN)_6_]_0.50_), Cs^+^ adsorption was observed. As shown in Fig. 8(a), definite desorption of Cu^2+^ was not observed at any *x*_obs_, even with *x*_obs_ = 0.50. To further evaluation for *x*_obs_ = 0.50, expanded shaking time of 72 h was employed for investigation, as shown in Fig. 8(b) and (c). The [Fe(CN)_6_] vacancy ratio, *x*, was almost constant during the adsorption process, indicating no desorption of metal cations. In contrast, the adsorption amount of Cs increased as the shaking time proceeded. The result showed that Cs-adsorption at *x*_obs_ = 0.50 was slower than at the other *x*.

Although there was a reported observation of Cu elution during Cs-adsorption took a long time (more than six months), we could not find a definite amount of Cu elution in three days, as shown in Fig. 8(a). Fig. 8(c) also shows no degradation of the crystal structure. In the usual adsorption processes in an appropriate period of less than three days, the contribution of ion-exchange between Cu^2+^ and Cs^+^ was rather small. The pH of the aqueous solution after Cs-adsorption test with KCF0.20 (*x*_obs_ = 0.50) shifted from 7.0 to 5.6, although the pH was almost unchanged with the use of KCF0.00 (*x*_obs_ = 0.25) and KCF 0.33 (*x*_obs_ = 0.33). From these results, we concluded that the mechanism of the initial stage of Cs-adsorption of KCuHCF with *x*_obs_ = 0.50 occurred by H^+^–Cs^+^ ion exchange as in insoluble Prussian blue,^8,35^ but this process was dominant only when the implemented amount of K^+^ was small, *e.g.*, *x* > 0.40.

We also evaluated the Cs position in the crystal after adsorption by Rietveld analysis with XRD patterns for *x*_obs_ = 0.33 and *x*_obs_ = 0.50, where the adsorption mechanisms were exchange with K^+^ and that with H^+^, respectively. Independent of *x*, the adsorption site was at the interstitial site. This was the same as the case of NH_4_^+^ adsorption by KCuHCF.^34^ The details of the derivation are mentioned in Section SI2 (ESI†).

### 3.4. Percolation model for capacity analysis

**Fig. 9:**
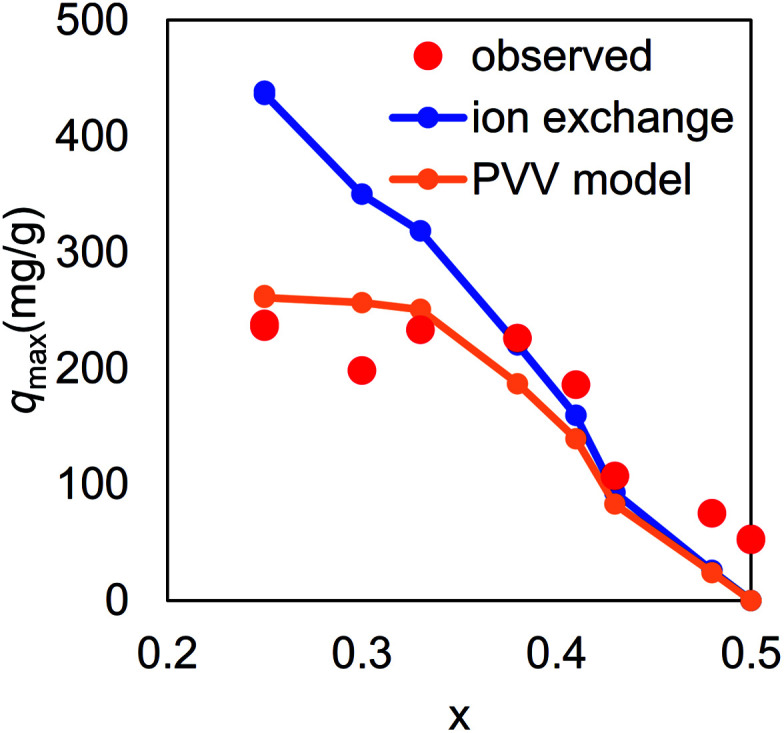
Cs-maximum capacity of KCuHCF with various vacancy ratios, *x*. Red circles indicate observed values, the blue line represents a theoretical one calculated with the assumption that all the K^+^ in KCuHCF exchanged with Cs^+^, and the orange line was derived from numerical analysis based on the PVV model.

The final question for the adsorption mechanism was the lower maximum capacity than that expected from the amount of K^+^ in KCuHCF at *x* < 0.38. As shown in Fig. 9, the observed maximum capacity was drastically smaller than that expected from the simple ion-exchange mechanism between K^+^ and Cs^+^, indicating that part of K^+^ in the crystal could not be exchanged with Cs^+^. In fact, the chemical composition of KCF0.00 (*x*_obs_ = 0.25) after the adsorption test was K_0.46_Cs_0.57_Cu[Fe(CN)_6_]_0.74_·2.4H_2_O, indicating that only 56% of K^+^ was replaced with Cs^+^.

**Fig. 10:**
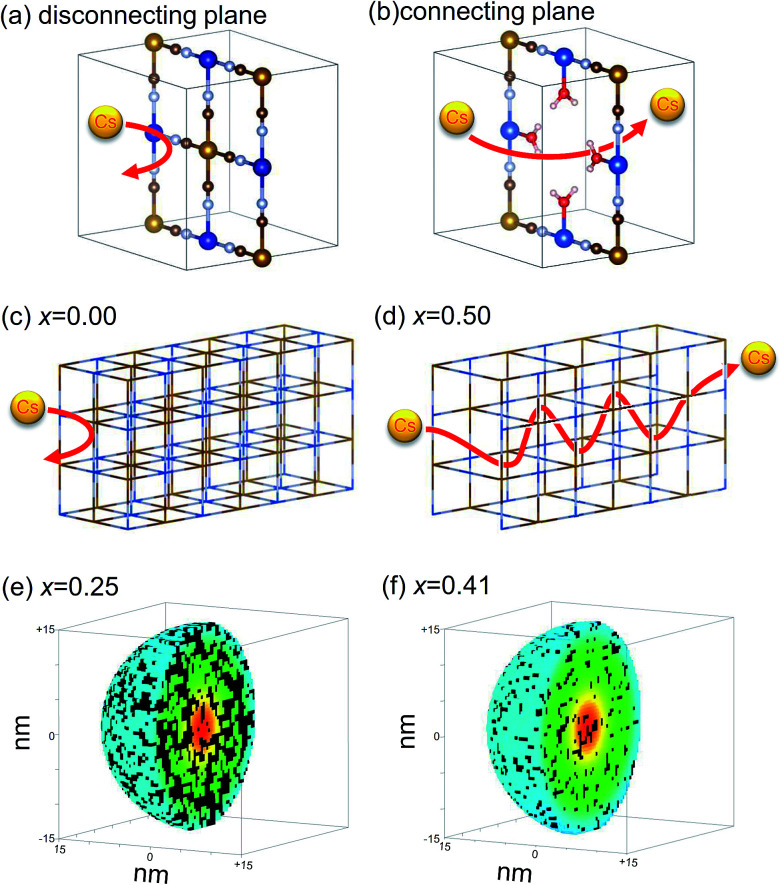
Schematic view of PVV model: schematic view of Cs migration through the crystal. (a) In a perfect lattice, Cs^+^ cannot pass without vacancy, but (b) can at a vacancy. No migration of Cs^+^ cation in the perfect KCuHCF lattice with *x* = 0 (c), and free migration with *x* = 0.50 (d). The results of numerical simulations of percolation profiles for KCuHCF nanoparticle at *x* = 0.25 (e) and at *x* = 0.41 (f) for 30 nm diameters. The coloured sites and black ones represent connected interstitial sites from the nanoparticle surface and the disconnected ones, respectively.

To unveil the open question, we propose a “percolation *via* vacancies (PVV)” model, where we hypothesize that Cs^+^ cation could not migrate in the perfect lattice of KCuHCF, but could do through [Fe(CN)_6_]^4−^ vacancies, schematically shown in Fig. 10(a) and (b). The Cs^+^ cation could pass through “the wall” between the adjacent interstitial sites only at a [Fe(CN)_6_]^2−^ vacancy (Fig. 10(b)), and could not do so through “the complete wall” without vacancies (Fig. 10(a)).

Our hypothesis is also energetically reasonable. The activation energy to overcome the complete wall for an alkali cation is calculated as the energy difference between the alkali cation at the interstitial site (0.25, 0.25, 0.25) and that in the wall (0.25, 0.50, 0.25) without vacancy, where the coordinates represent the position in the unit cell shown in Fig. 1. The possibility for an alkali cation to overcome the complete wall, *P*_Cs_, is calculated from Arrhenius' equation:

6 *P*_A^+^_ = A exp[−*E*_a_(A^+^)/*k*_B_*T*],

where A, *E*_a_(A^+^), *k*_B_, and *T* represent the pre-exponential factor, the activation energy for A^+^ to overcome the complete wall (A^+^ = Cs^+^ or K^+^), Boltzmann's constant, and the temperature, respectively. The pre-exponential factor is assumed to be the same for both Cs^+^ and for K^+^. The ratio of the possibility *P*_Cs^+^_/*P*_K^+^_ is obtained as

7 *P*_Cs^+^_/*P*_K^+^_ = exp[(*E*_a_(Cs) − *E*_a_(K))/*k*_B_*T*].

The activation energies were estimated by Moritomo and Tanaka using *ab initio* total energy calculation.^53^ For Cs^+^ and K^+^, the activation energies were estimated at ∼2.3 eV and ∼0.6 eV per cation, respectively. With these values, *P*_Cs^+^_/*P*_K^+^_ was calculated to 3.6 × 10^−16^ at *T* = 300 K, implying that the possibility of overcoming the complete wall for Cs^+^ was extremely smaller than that of K^+^ at room temperature.

To clarify the meanings of our hypothesis in the adsorption process, we describe two typical examples, shown in Fig. 10(c) and (d). In the perfect lattice of K_2_Cu[Fe(CN)_6_], without vacancies (*x* = 0.00) (shown in Fig. 1(a)), Cs^+^ could not penetrate the crystal at all (Fig. 10(c)). In contrast, in the crystal of Cu[Fe(CN)_6_]_0.5_, shown in Fig. 1(c), with periodic [Fe(CN)_6_] vacancy alignment, Cs^+^ could freely migrate in the crystal Fig. 10(d). As a result, only K^+^ located at interstitial sites connected to the nanoparticle surface *via* vacancy network could be used for ion-exchange with Cs^+^, because Cs^+^ adsorbed and K^+^ desorbed through the nanoparticle surface. This consideration belongs to the concept of percolation. Actually, the migration path would be complicated because of the random distribution of vacancies in MHCFs,^47^ but the estimation of the amount of exchangeable K^+^s in the crystal was numerically possible.

The saturated capacity in the PVV model, *q*^PVV^_max_, is calculated *via* percolation analysis by

8 *q*^PVV^_max_ [mg g^−1^] = *yρ*_C_ × 1000*M*,

where *y*, *ρ*_C_, and *M* represent the molar ratio of K in the chemical composition, the ratio of interstitial sites connected to the nanoparticle surface *via* the vacancy network, and the ratio of atomic weight of Cs to the molecular weight of KCuHCF.

Fig. 9 shows *q*^PVV^_max_ as a function of *x*, calculated with *ρ*_C_ derived from a numerical simulation of spherical nanoparticles in 30 × 30 × 30-unit cells, including ∼105 000 interstitial sites. The result clearly shows that the PVV model reproduced the observed *q*_max_. In the case of *x* = 0.25, the ratio of exchangeable sites was estimated to be 60%, comparable with the observed one, 56%, described above. Fig. 10(e) and (f) show the distribution of interstitial sites connected to the nanoparticle surfaces *via* vacancies. Both the connected sites and disconnected ones seemed to be randomly distributed in the nanoparticles.

It should be emphasized that our numerical simulation required no fitting parameters. Only by setting structural parameters, *i.e.*, the particle size and the [Fe(CN)_6_] vacancy ratio, the ratio of exchangeable sites was automatically determined. In addition, the result was independent of the nanoparticle size when the size was above a certain level.

Thus, our PVV model explains the suppression of Cs-adsorption on KCuHCF in the range of low *x*. In addition, the model also gave a solution for the difference in Cs-adsorption property between insoluble PB and the soluble one, described in Section 1. As described before, the chemical compositions of insoluble PB and the soluble one are (NH_4_)_0.70_Fe_1.10_[Fe(CN)_6_]·1.7H_2_O and Fe_4_[Fe(CN)_6_]_3_, respectively.^8^ Although soluble PB had more exchangeable cations, NH_4_^+^, only low Cs-adsorption was observed. This behavior can be understood through the low vacancy ratio of soluble PB, *x* = 0.09, where the ratio of exchangeable sites was estimated to be only 4% by the PPV model.

## Conclusions

We confirmed that the stable range of [Fe(CN)_6_] vacancy ratio was 0.25 < *x* < 0.50 for an appropriate time for Cs-adsorption. In this range, the relationships between the chemical composition factors *x*, *y*, and *z* in K_*y*_Cu[Fe(CN)_6_]_1−*x*_·*z*H_2_O were obtained as *y* = 2 − 4*x* and *z* = 10*x*, indicating complete dehydration of K^+^ in the MHCF crystal. We concluded that the complete dehydration of alkali cations was the reason for the excellent selectivity of Cs in adsorption.

Cs-adsorption by KCuHCF was governed by three mechanisms: (a) mainly ion-exchange between Cs^+^ and K^+^ (K-exchange), (b) percolation of Cs^+^ cations through vacancy sites from the surface, described by the PVV model at smaller *x*, and (c) proton-exchange with Cs^+^ at the range of low K^+^ incorporation. The main contribution determining the saturation capacity was from K^+^–Cs^+^ ion exchange. However, in the range of low concentration of [Fe(CN)_6_] vacancies, the PVV model governed. The PVV mechanism is applicable to other MHCFs. For example, the low Cs-adsorption of soluble PB is explained by the model.

## Conflicts of interest

There are no conflicts to declare.

## Supplementary Material

RA-008-C8RA06377J-s001

## References

[cit1] Hashimoto S., Ugawa S., Nanko K., Shichi K. (2012). Sci. Rep..

[cit2] Environmental consequences of the Chernobyl accident and their remediation: Twenty years of experience. Report of the Chernobyl Forum Expert group ‘Environment’, IAEA, 2006

[cit3] Haas A. P. (1993). Sep. Sci. Technol..

[cit4] Loos-Neskovic C., Fedoroff M. (1988). React. Polym., Ion Exch., Sorbents.

[cit5] Parajuli D., Takahashi A., Noguchi H., Kitajima A., Tanaka H., Takasaki M., Yoshino K., Kawamoto T. (2016). Chem. Eng. J..

[cit6] Yasutaka T., Tsuji H., Kondo Y., Suzuki Y., Takahashi A., Kawamoto T. (2015). J. Nucl. Sci. Technol..

[cit7] Harjula R., Lehto J., Paajanen A., Tusa E., Yarnell P. (2004). React. Funct. Polym..

[cit8] Ishizaki M., Akiba S., Ohtani A., Hoshi Y., Ono K., Matsuba M., Togashi T., Kananizuka K., Sakamoto M., Takahashi A., Kawamoto T., Tanaka H., Watanabe M., Arisaka M., Nankawa T., Kurihara M. (2013). Dalton Trans..

[cit9] Lee K.-M., Kawamoto T., Minami K., Takahashi A., Parajuli D., Kido G., Yoshino K., Tanaka H. (2016). RSC Adv..

[cit10] Takahashi A., Minami N., Tanaka H., Sue K., Minami K., Parajuli D., Lee K.-M., Ohkoshi S., Kurihara M., Kawamoto T. (2015). Green Chem..

[cit11] Torad N. L., Hu M., Imura M., Naito M., Yamauchi Y. (2012). J. Mater. Chem..

[cit12] Chen G.-R., Chang Y.-R., Liu X., Kawamoto T., Tanaka H., Kitajima A., Parajuli D., Takasaki M., Yoshino K., Chen M., Lo Y., Lei Z., Lee D. (2015). Sep. Purif. Technol..

[cit13] Abusafa A., Yücel H. (2002). Sep. Purif. Technol..

[cit14] Ikarashi Y., Mimura H., Nakai T., Niibori Y., Ishizaki E., Matsukura M. (2014). J. Ion Exch..

[cit15] Borai E. H., Harjula R., Malinen L., Paajanen A. (2009). J. Hazard. Mater..

[cit16] Lamare V., Dozol J.-F., Fuangswasdi S., Arnaud-Neu F., Thuéry P., Nierlich M., Asfari Z., Vicens J. (1999). J. Chem. Soc., Perkin Trans. 2.

[cit17] Dozol J. F., Simon N., Lamare V., Rouquette H., Eymard S., Tournois B., De Marc D., Macias R. M. (1999). Sep. Sci. Technol..

[cit18] Casnati A., Pochini A., Ungaro R., Ugozzoli F., Arnaud F., Fanni S., Schwing M.-J., Egberink R. J. M., de Jong F., Reinhoudt D. N. (1995). J. Am. Chem. Soc..

[cit19] Duignan M. R., Nash C. A. (2010). Sep. Sci. Technol..

[cit20] Aguila B., Banerjee D., Nie Z., Shin Y., Ma S., Thallapally P. K. (2016). Chem. Commun..

[cit21] Seino S., Kawahara R., Ogasawara Y., Mizuno N., Uchida S. (2016). Angew. Chem., Int. Ed..

[cit22] Celestian A. J., Kubicki J. D., Hanson J., Clearfield A., Parise J. B. (2008). J. Am. Chem. Soc..

[cit23] Anthony R. G., Dosch R. G., Gu D., Philip C. V. (1994). Ind. Eng. Chem. Res..

[cit24] Kaye S. S., Long J. R. (2005). J. Am. Chem. Soc..

[cit25] Takahashi A., Tanaka H., Parajuli D., Nakamura T., Minami K., Sugiyama Y., Hakuta Y., Ohkoshi S., Kawamoto T. (2016). J. Am. Chem. Soc..

[cit26] Thallapally P. K., Motkuri R. K., Fernandez C. A., McGrail B. P., Behrooz G. S. (2010). Inorg. Chem..

[cit27] Motkuri R. K., Thallapally P. K., McGrail B. P., Ghorishi S. B. (2010). CrystEngComm.

[cit28] Boudjema L., Mamontova E., Long J., Larionova J., Guari Y., Trens P. (2017). Inorg. Chem..

[cit29] Krap C. P., Balmaseda J., Zamora B., Reguera E. (2010). Int. J. Hydrogen Energy.

[cit30] Neff V. D. (1978). J. Electrochem. Soc..

[cit31] Lee K.-M., Tanaka H., Takahashi A., Kim K. H., Kawamura M., Abe Y., Kawamoto T. (2015). Electrochim. Acta.

[cit32] Takachi M., Matsuda T., Moritomo Y. (2013). Appl. Phys. Express.

[cit33] Xie X., Ye M., Liu C., Hsu P., Criddle C. S., Cui Y. (2015). Energy Environ. Sci..

[cit34] Parajuli D., Noguchi H., Takahashi A., Tanaka H., Kawamoto T. (2016). Ind. Eng. Chem. Res..

[cit35] Guo T. , Yun S., Li H., Zhu X. and Gao X., in Proceedings of the 2016 2nd International Conference on Architectural, Civil and Hydraulics Engineering (ICACHE 2016), Atlantis Press, Paris, France, 2016, p. 272

[cit36] Ayrault S., Jimenez B., Garnier E., Fedoroff M., Jones D., Loos-Neskovic C. (1998). J. Solid State Chem..

[cit37] Loos-Neskovic C., Ayrault S., Badillo V., Jimenez B., Garnier E., Fedoroff M., Jones D. J., Merinov B. (2004). J. Solid State Chem..

[cit38] Dwivedi C., Kumar A., Singh K. K., Juby A. K., Kumar M., Wattal P. K., Bajaj P. N. (2013). J. Appl. Polym. Sci..

[cit39] Jang S.-C., Haldorai Y., Lee G.-W., Hwang S.-K., Han Y.-K., Roh C., Huh Y.S. (2015). Sci. Rep..

[cit40] Chen R., Asai M., Fukushima C., Ishizaki M., Kurihara M., Arisaka M., Nankawa T., Watanabe M., Kawamoto T., Tanaka H. (2014). J. Radioanal. Nucl. Chem..

[cit41] Nilchi A., Saberi R., Moradi M., Azizpour H., Zarghami R. (2011). Chem. Eng. J..

[cit42] Hwang K. S., Park C. W., Lee K.-W., Park S.-J., Yang H.-M. (2017). Colloids Surf., A.

[cit43] Gaur S. (1996). J. Chromatogr. A.

[cit44] Yasutaka T., Miyazu S., Kondo Y., Tsuji H., Arita K., Hayashi S., Takahashi A., Kawamoto T., Aoyama M. (2016). J. Nucl. Sci. Technol..

[cit45] Zadronecki M., Linek I. A., Stroka J., Wrona P. K., Galus Z. (2001). J. Electrochem. Soc..

[cit46] National Effluent Standards, Ministry of the Environment, Japan, 2015

[cit47] Grandjean F., Samain L., Long G. J. (2016). Dalton Trans..

[cit48] Ghosh S. N. (1974). J. Inorg. Nucl. Chem..

[cit49] Ayrault S., Jimenez B., Garnier E., Fedoroff M., Jones D., Loos-Neskovic C. (1998). J. Solid State Chem..

[cit50] Eberl D. D. (1980). Clays Clay Miner..

[cit51] Housecroft C. E., Brooke Jenkins H. D. (2017). RSC Adv..

[cit52] Zong Y., Zhang Y., Lin X., Ye D., Qiao D., Zeng S. (2017). RSC Adv..

[cit53] Moritomo Y., Tanaka H. (2013). Adv. Condens. Matter Phys..

