# Supplementary material for: Unveiling Cs-adsorption mechanism of Prussian blue analogs: Cs+-percolation via vacancies to complete dehydrated state
Source: RSC Adv. 2018 Oct 10;8(61):34808–16. doi: 10.1039/c8ra06377j (PMC9087018; doi:10.1039/c8ra06377j)
Supplement: RA-008-C8RA06377J-s001 [file RA-008-C8RA06377J-s001.pdf]

Electronic Supplementary Material (ESI) for RSC Advances.
This journal is © The Royal Society of Chemistry 2018

Supporting information

Unveiling Cs-adsorption mechanism of Prussian blue analogs: Cs^+-percolation via vacancies to complete dehydrated state

Akira Takahashi[1], Hisashi Tanaka[1], Kimitaka Minami[1], Keiko Noda[1], Manabu Ishizaki[2], Masato Kurihara[1,2], Hiroshi Ogawa[2], and Tohru Kawamoto[1*]

1 Nanomaterials Research Institute, AIST, 1-1-1 Higashi, Tsukuba 305-8565, Japan.

2. Department of Material and Biological Chemistry, Faculty of Science, Yamagata University, 1-4-12 Kojirakawa-machi, Yamagata 990-8560, Japan,

3 Research Center for Computational Design of Advanced Functional Materials, AIST, 1-1-1 Umezono, Tsukuba 305-8568, Japan.

SI 1 Shaking time dependence of adsorption amount

The shaking time dependence of Cs adsorption was evaluated by batch adsorption experiments. sample powder (10 mg) of KCF0.33, KCF0.38, KCF0.41, KCF0.44, and KCF-0.50, were added to 10 mL $CsNO_3$ aqueous solution with Cs-concentration 300 mg/L, then shaken at 25 °C using a shaker for 1, 3, 16, and 66 h. After shaking, the residue was separated from the supernatant. The concentration of Cs in the supernatant was evaluated by ICP-MS. The amount of Cs adsorbed was calculated using eq (1). The Cs-adsorption amounts showed no critical difference for 1 to 66 h, as shown in Fig SI.1.

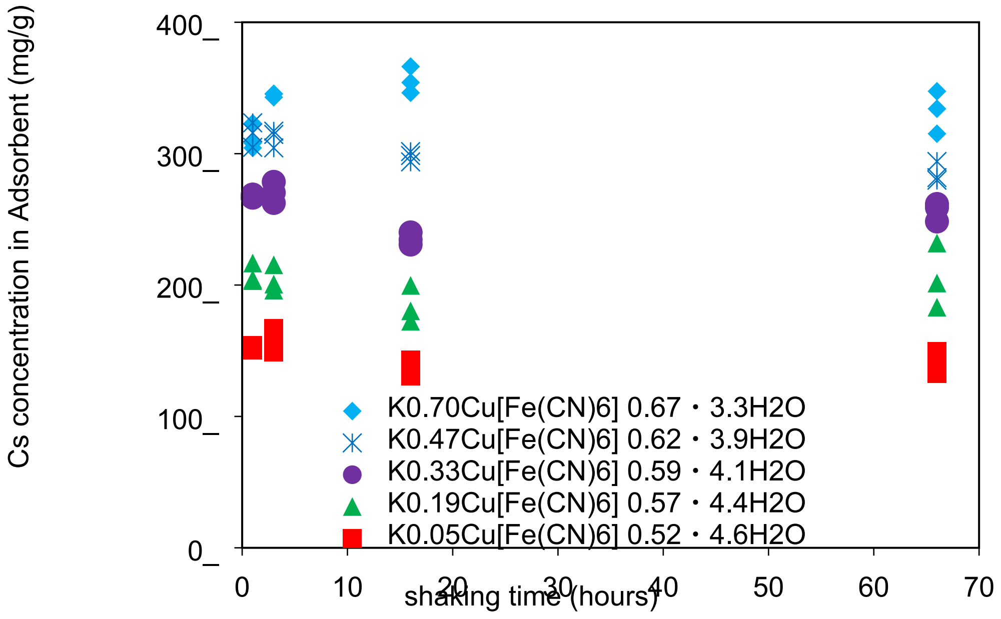

Figure SI1. Shaking time dependence for Cs-adsorption amount.

SI 2 Rietveld refinement

The crystal structures of samples adsorbing Cs^+ were evaluated by Rietveld refinement of the powder XRD pattern using TOPAS 5 (Bruker AXS Inc.). Two different compositions of CuHCFs: KCF-0.33, and KCF-0.80 were evaluated before and after Cs-adsorption.

The sample (40 mg) was added to 40 mL $CsNO_3$ aqueous solution with Cs-concentration 1000 mg/L, then shaken at 25 ˚C using a shaker for 100 min (for KCF-0.33) or 24 h (for KCF-0.80). After shaking, the sample powders were filtered by 0.45 micrometer membrane filter and dried at 60 ˚C.

Powder XRD patterns of KCuHCF-NPs before and after Cs-adsorption were obtained, as shown in Fig. SI2. In the case of KCuHCF-NPs before Cs-adsorption, the samples were mixed with an angle standard silicon powder (NIST 640e) before powder XRD measurement. In the analysis, bond length is assumed to be 1.16 Å.

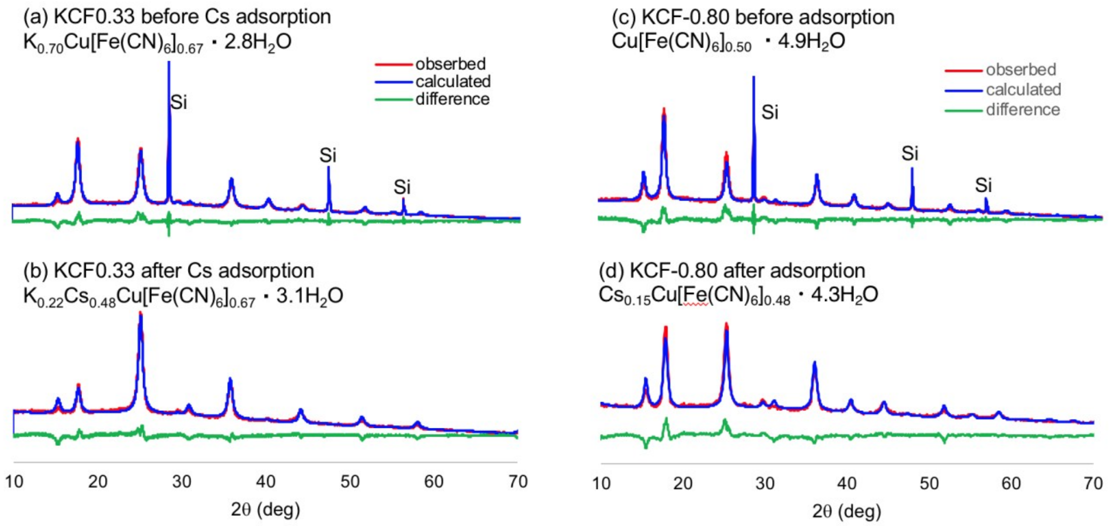

Figure SI2. Rietveld refinement for evaluating Cs position.

Table SI1. Atomic position of $K_{0.70}Cu[Fe(CN)_6]_{0.67}{\cdot}2.8H_2O$ obtained by Rietveld refinement.

atom	x	y	z	B_{eq}
K	0.25000	0.25000	0.25000	20.00
Cu	0.00000	0.00000	0.00000	19.99
N	0.21747	0.00000	0.00000	19.22
C	0.33347	0.00000	0.00000	5.484
Fe	0.50000	0.00000	0.00000	19.99
O(v)	0.22212	0.00000	0.00000	16.86
O(i)	0.25000	0.25000	0.25000	20.00

SI 3. Langmuir plot for Cs adsorption by KCuHCF

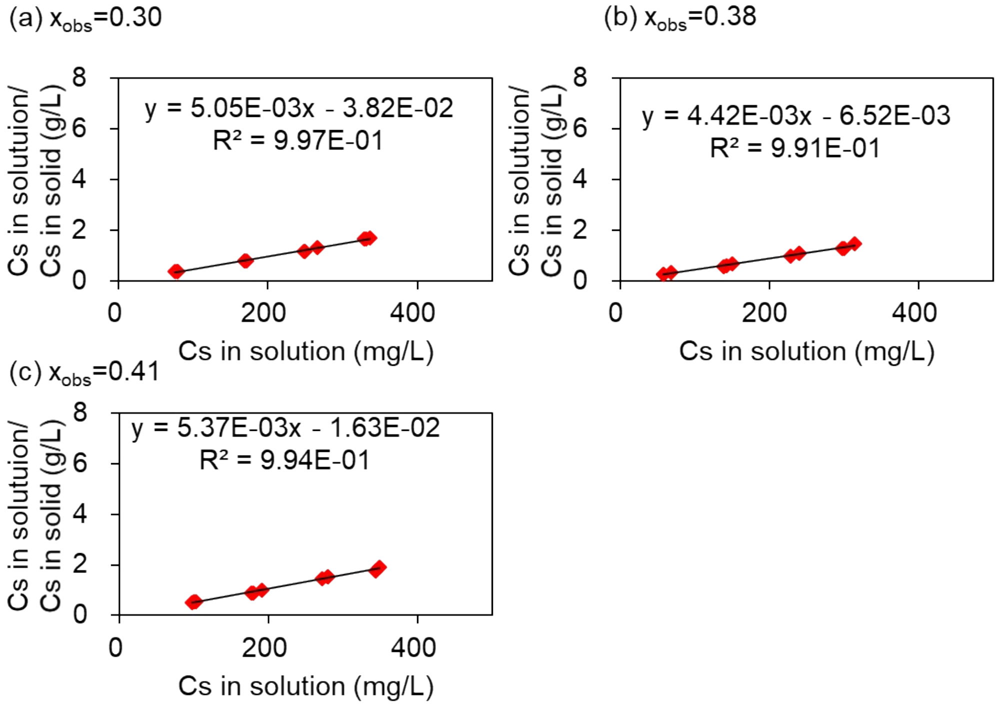

Figure SI3. Langmuir plot for Cs adsorption by KCuHCF with varying *x* (not shown in Fig. 4). The black line represents the curve fit by the Langmuir equation.

SI 4. Detail of the percolation analysis

The evaluation of the saturated capacity based on the PVV model was done numerically. Before the model applied to KCuHCF, a two-dimensional PPV model is shown for simple explanation of our concept. Figure SI4 represents the schematic view of a two-dimensional PPV model, where the interstitial sites, blue or red circles, indicate the positions that can be occupied by K^+ or Cs^+. The black or white circles indicate the metal site. In this model, the site in the nanoparticle is only considered. The metal sites are partially occupied (black), and the others are the vacancies (white). Each interstitial site was judged whether it was the connected (blue) or disconnected (red) sites from the nanoparticle surface. In the judgement of the connectivity, the connecting path represented with white line between the adjacent interstitial sites. Namely, the adjacent interstitial sites sandwiched the vacancy metal site (white) is connected. If an interstitial site is connected with the connection lines from the nanoparticle surface, the sites is judged as "connected from the surface". If not, it is "disconnected from the surface". Finally, the ρ_C in equation 8 in the main text is calculated as $n_c / (n_c + n_{dc})$, where n_c and n_{dc} represent the number of the connected and disconnected sites from the surface

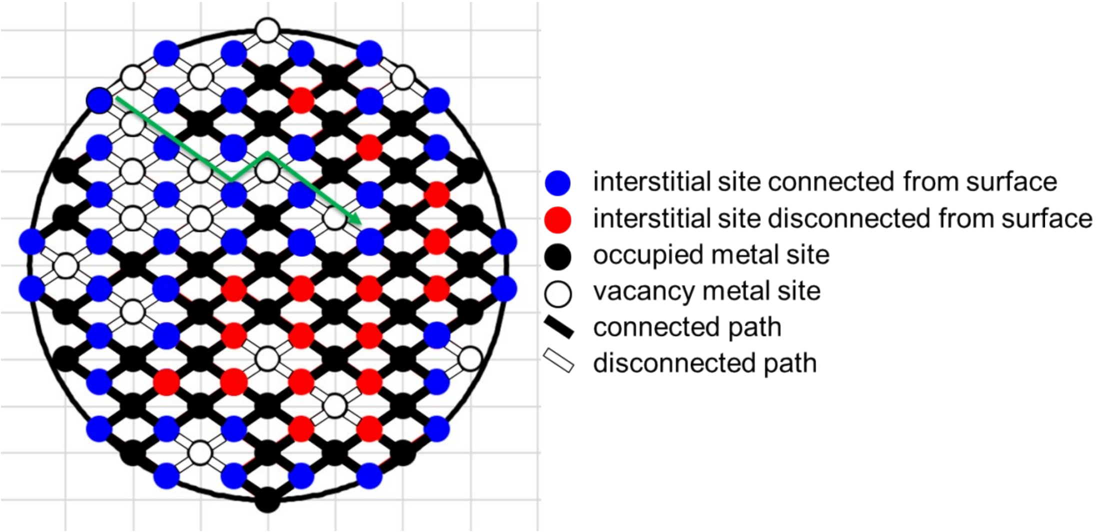

Figure SI4. Schematic view of a two-dimensional PPV model. The large circle represents a nanoparticle. Green arrow represents an example of the connecting path from the nanoparticle surface.

In the case of the three-dimensional lattice for KCuHCF, we calculated ρ_C as follows. At first, we prepare the simplified lattice consisting of the metallic site, Cu and Fe, schematically shown in Figure SI5, where Fe, Cu, and I represent the Fe-site, Cu-site and the interstitial site. Cs and K are located at interstitial sites. The $[Fe(CN)_6]$ vacancies are randomly located to adjust the ratio

of Cu/Fe to fit the chemical composition experimentally evaluated. when Fe-site is substituted with the vacancy, the adjacent interstitial site across the vacancy is considered as the "connected". For example, Fe(0,0,1) or Fe(0,1,0) in Figure SI5 is vacant, I(0,1,1) and I(1,1,1) is considered as connected. Figure SI6 shows the connection of the interstitial site in more realistic view. The eight interstitial sites surrounding the [$Fe(CN)_6$] vacancies are considered as "connected".

The next step is the construction of the nanoparticle model. In this model, we assume the lattice constant is 1.00 nm, because the lattice constant of KCuHCF were experimentally evaluated to be between 0.996 and 1.005 nm as shown in Table 1 in the main text. Finally, we count the interstitial site connected from the surface and that disconnected from the surface numerically, where the procedure is the same as the two-dimensional case described above.

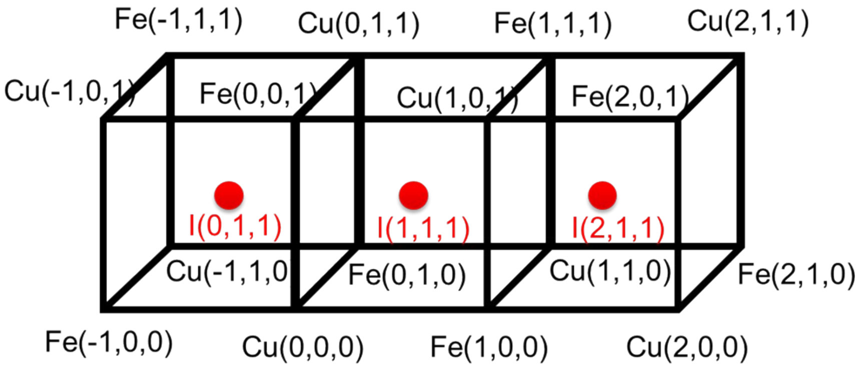

Figure SI5. Schematic view of the simplified lattice used in the numerical analysis for PVV model. Cu, Fe, and I represent the Cu-site, Fe-site and interstitial site, respectively.

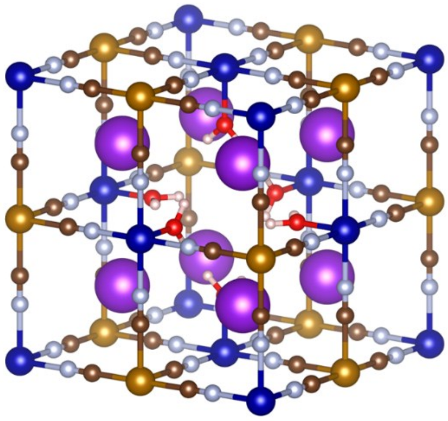

Figure SI6. Schematic view of the eight connected interstitial site via a [$Fe(CN)_6$] vacancy.
